# Patient-reported experience is associated with higher future revenue and lower costs of hospitals

**DOI:** 10.1007/s10198-023-01646-y

**Published:** 2023-12-09

**Authors:** Alice Giese, Rasheda Khanam, Son Nghiem, Thomas Rosemann, Michael M. Havranek

**Affiliations:** 1 University of Southern Queensland, Toowoomba, Australia; 2 Competence Center for Health Data Science, Faculty of Health Sciences and Medicine, University of Lucerne, Frohburgstrasse 3, Postfach, 6002 Lucerne, Switzerland; 3 Department of Health Economics, Wellbeing & Society, Australian National University, Canberra, Australia; 4 Institute of Primary Care, University of Zurich and University Hospital Zurich, Zurich, Switzerland

**Keywords:** Patient-reported experience, Hospitals, Financial performance, Elective patients, I10, I11, I15, I18

## Abstract

**Background:**

Despite the established positive association between patient experience and patient volume, the relationship between patient experience and the financial performance of hospitals has not been studied thoroughly.

**Methods:**

To investigate this relationship, we used longitudinal data from 132 Swiss acute-care hospitals from 2016 to 2019 to examine the associations between patient experience and the proportion of elective patients, revenue, costs, and profits of hospitals. To account for a potential time lag effect, we utilized annual patient experience data and employed multilevel mixed-effects regression modeling to investigate its association with the aforementioned financial performance indicators for the following year.

**Results:**

Data for private and public hospitals were analyzed both separately and in combination, to account for the different proportions of elective patients in these types of hospitals. The resulting mixed models, revealed that for each year studied, the previous year’s patient experience was positively associated with the current year’s proportion of elective patients (β = 0.09, *p* = 0.004, all hospitals) and revenue (β = 1789.83, *p* = 0.037, private hospitals only), and negatively associated with costs (β =  − 1191.13, *p* = 0.017, all hospitals); but not significantly associated with future profits (β = 629.12, *p* = 0.240, all hospitals).

**Conclusions:**

This analysis showed that better patient experience is associated with a higher proportion of elective patients, greater revenue, and lower costs. Our findings may assist hospital managers and regulators in identifying strategies to increase revenue and reduce costs.

**Supplementary Information:**

The online version contains supplementary material available at 10.1007/s10198-023-01646-y.

## Introduction

Hospitals are under growing financial pressure due to reimbursement constraints and a shortage of qualified staff; a situation that has been further exacerbated by the consequences of the COVID-19 pandemic. In confronting this challenge, hospitals can attempt to increase revenue and/or reduce costs. On the one hand, they can generate additional revenue by increasing patient volume [1], expanding their specialized services (i.e., elective procedures such as surgery, radiology, cancer treatment, cardiology, or orthopedics) [2], focusing on patients with supplemental insurance that generates additional income [3], and offering services with high demand and limited competition [4]. On the other hand, hospitals can enhance their profitability through more efficient operations and cost management [5].

Most revenue-oriented strategies to enhance profitability focus on elective patients that can be attracted by marketing, loyalty initiatives, and positive reputation; all of which are supported by positive patient experience [6–10]. However, cost-management strategies to increase the bottom line also benefit from better patient experience, since this results in fewer complaints along with less frequent malpractice claims and liability cases [11]. Despite this, only a few existing studies have investigated the relationship between patient experience and the financial performance of hospitals, with mixed results [12, 13].

Patient experience has been found to positively influence patient loyalty [6, 8, 10, 14] and word-of-mouth recommendations [7, 9]. For instance, Richter and Muhlestein [13] showed that higher inpatient satisfaction (which is closely related to patient experience; see Methods section) was associated with increased profitability through higher operating margins. However, Richter and Muhlestein [13] neglected to control for potential confounders [15]. Other researchers have reported associations between patient experience and hospital financial performance, but they only focused on countries with incentives for better patient experience [16]. In addition, all previous studies have examined related measures such as revenue or profits separately, rather than considering these financial performance indicators collectively [13, 16, 17].

Thus, the present study aimed to more comprehensively examine the relationship between patient experience and hospital financial performance with regard to revenue, costs, and profits. Using multilevel (hierarchical) mixed-effects regression modeling on a longitudinal dataset from all Swiss acute-care hospitals, we tested the hypotheses that the average patient experience of hospitals for a given year is associated with (1) a higher proportion of elective patients, (2) higher revenue, (3) lower costs, and (4) higher profits in the following year.

## Methods

### Data sources and national setting

We used publicly available national administrative data on the aggregated financial performance of Swiss hospitals from the Swiss Federal Statistical Office, for the period 2016–2019 [18]. To this dataset, we linked patient experience results collected via a validated survey [19], which is published annually by the Swiss National Association for Quality Development in Hospitals and Clinics [20]. From the original sample of 139 Swiss acute-care hospitals, seven were excluded before performing the analyses: one because of missing revenue data, two because of their incomparably small size (i.e., regarding the number of beds), and four due to an unrealistically high cost/revenue ratio. This gave a final sample of 132 acute-care hospitals for subsequent analysis.

Of the 132 hospitals, 82 (62.1%) are publicly and 50 (37.9%) are privately owned. As explained above, elective patients are crucial for the investigation of patient experience and financial performance, because emergency patients cannot choose their hospital freely due to the inherent circumstances of their admission. Private hospitals tend to have an (often considerably) higher proportion of elective patients [21], and this is also observed in Switzerland (see Online Appendix, Fig. S1). To account for this, we subdivided hospitals according to their ownership (i.e., publicly or privately owned), as has been done previously [22], and therefore performed all analyses independently on three samples: a sample of all 132 public and private hospitals, a sample including only the 82 public hospitals, and a sample including only the 50 private hospitals.

In Switzerland, health insurance is compulsory for all residents and patients have free hospital choice, which allows for patient loyalty [23]. In addition, various factors lead to competition among Swiss hospitals: the prospective payment system based on diagnosis-related groups (DRGs), the high hospital density, and more lucrative supplementary insured patients [24]. However, Swiss hospitals are not currently (as of 2023) offered any financial incentives that reward better patient experience. Thus, the country provides an attractive setting in which to investigate associations between patient experience and financial performance, independently of additional incentives.

### Variables

#### Dependent variables

In accordance with previous research, we measured hospitals’ financial performance by aggregated: (1) net acute-care inpatient revenues, (2) net acute-care inpatient operating expenses [1], and (3) acute-care inpatient profits [25]. To ensure the comparability of hospitals, net operating expenses excluded depreciation and amortization, other rental expenses, and imputed interests on fixed assets. To account for other heterogeneity among hospitals (e.g., differences in size), we standardized the financial measures by the sum of their DRG cost weights (i.e., the total case mix of the hospitals), which is a routinely used procedure to enable comparison of hospital financial data [26]. Assumptions of normality were rejected for all three financial parameters based on visual identification of excessive skewness and kurtosis, and the Shapiro–Wilk test also rejected the parameters’ normality (*p* < 0.001). Financial data were assessed in Swiss francs (CHF), which is convertible into US dollars (USD) at an exchange rate of 1.08 (CHF 1 = USD 1.08) and into euros (EUR) at an exchange rate of 1.01 (CHF 1 = EUR 1.01), as of February 2023. In addition to our primary outcomes concerning hospital financial performance, we also investigated the proportion of elective patients as a secondary outcome of interest. This was calculated as the complementary probability of the proportion of emergency admissions per hospital. We deliberately focused on the relative proportion of elective patients rather than the absolute number of elective patients because the latter is highly dependent on/confounded by the size of the hospitals.

#### Independent variables

Whereas patient satisfaction refers to the overall satisfaction of a patient with a healthcare encounter, and describes how well the patient’s needs and expectations were met [27], patient experience is viewed as a more objective and therefore more accurate measure [27]. The ANQ assesses patient experience using five questions (Q1–Q5) relating to: (1) their overall satisfaction with the quality of care, (2) the ability to ask questions, (3) the clarity of answers, (4) the explanations on medications, and (5) the discharge process. The five exact questions can be found in the Online Appendix (Table S1).

The ANQ [20] has reported the results for these five questions annually since 2016, as five separate mean values for each hospital. For the purposes of the present analyses, the average of the five mean values was used. Although the ANQ publishes the results under the term “patient satisfaction,” the majority of the five questions refer to individual patient experience and will accordingly be referred to hereafter as “patient experience.” The five-point verbal scale that is used has previously demonstrated excellent internal consistency (Cronbach’s alpha: 0.96) [28].

#### Covariates

To account for the potential influence of covariates in our analyses, we included variables that are known to affect hospitals’ financial performance, according to the literature. Because patient volume and geographic location have previously shown associations with both revenue and costs, we used the hospitals’ number of discharges [29] and location (urban vs. rural) [30] as covariates. To capture patient complexity and its potential influence on revenue and costs, we included hospitals’ case-mix indices (CMIs) as proposed by Richter and Muhlestein [13]. Finally, as the proportion of patients with supplemental health insurance (SI) has a significant impact on revenue, this was also accounted for [31].

### Statistical analyses

Basic statistical descriptors (mean, *M*, and standard deviation, *SD*) were calculated for the variables, and Cronbach’s alpha was used to evaluate the internal consistency of the patient experience survey. After careful examination of the explanatory variables, we employed multilevel (hierarchical) mixed-effects regression modeling to examine the influence of the time-lagged patient experience for each year from 2016 to 2018 (PE_Lag), on hospitals’ proportion of elective patients, revenue, costs, and profits for the following year (2017–2019).

The model was defined with fixed effects for the independent variable and covariates as defined above: PE_Lag, SI, Location, CMI, and Discharges. The random effects (ζ) were applied to the hospitals’ identification number (ID, *i*), and time (year, *t*) was treated as a repeated effect (η). The base model to explain the dependent variables (*dep.var*_*it*_*,* consisting of hospitals’ proportion of elective patients, revenue, costs, and profits) was defined by the following equation:

1 $$ dep.{\text{var}}_{it} = \left( {\beta_0 + \zeta_i } \right) + \beta_1 \cdot PE\_Lag_{it} + \beta_2 \cdot SI_{it} + \beta_3 \cdot Location_{it} + \beta_4 \cdot CMI_{it} + \beta_5 \cdot Discharges_{it} + \eta_t + \varepsilon_{it} $$

for *i* = 1, …, *N*_*hosp*_, and *t* = 1, …, *T*_*year*_, where

- PE*_*Lag_*it*_ = patient experience from previous year (time-lagged);
- *SI*_*it*_ = proportion of patients with supplemental health insurance;
- *Location*_*it*_ = location of hospitals (binary, rural vs. urban);
- *CMI*_*it*_ = case-mix index of hospitals;
- *Discharges*_*it*_ = number of acute-care discharges of hospitals;
- η_t_ = time effect;
- ε_it_ = error term; and
- ζ_i_ = random effect applied to the intercept of the model.

Regression diagnostics including the tolerance test for multicollinearity, its reciprocal variance inflation factors, and the Akaike information criterion (AIC), were used to assess the model’s goodness of fit. For all statistical analyses, the software IBM SPSS Statistics 27 was used, and results were considered significant where *p* < 0.05.

## Results

### Description of the data

Data from 132 Swiss acute care hospitals were used in the analysis. Of these 132 hospitals, five (3.8%) were university hospitals (i.e., academic teaching hospitals), 39 (29.5%) were large central general hospitals, 57 (43.2%) were medium-sized and small regional general hospitals, and 31 (23.5%) were surgical and other specialty hospitals. Of these hospitals, 47 (35.6%) were located in urban areas (defined as cities having > 35,000 citizens) and 85 (64.4%) in rural areas. The average number of acute-care patients per year per hospital was 9975 (*SD* = 10,875), and these were treated using an average of 180 beds per hospital (*SD* = 219).

On average, 24.0% (*SD* = 18.4%) of patients had supplemental health insurance, although this differed between public (16.8%, *SD* = 7.6%) and private (35.2%, *SD* = 23.8%) hospitals. Overall, 62.4% (*SD* = 23.9%) of patients had been admitted electively (i.e., not as an emergency), with private hospitals reporting significantly more scheduled admissions (86.0% elective patients, *SD* = 5.0%) compared with public hospitals (46.8% elective patients, *SD* = 15.0%). In addition, private hospitals had a slightly higher CMI (1.02, *SD* = 0.21) than public hospitals (0.95, *SD* = 0.24). By hospital type, the CMI was highest among university hospitals (1.40, *SD* = 0.15), followed by surgical and other specialty hospitals (1.07, *SD* = 0.20), and the lowest CMI was found in regional general hospitals (0.88, *SD* = 0.20).

Mean standardized costs were CHF 9250 (*SD* = 1375; range 1539–18,193), mean standardized revenue was CHF 9531 (*SD* = 1583; range 2235–23,311), and mean standardized profits were CHF 280 (*SD* = 1519; range  − 7829–11,247).

Overall, patient experience was found to be high (4.36, *SD* = 0.14), and all five individual items of the patient experience survey had mean ratings above 4.0 (see Online Appendix, Table S2). Cronbach’s alpha was 0.87 based on our sample. However, private hospitals displayed a statistically significantly (*p* < 0.001, Cohen’s *d* = 0.88) higher number of satisfied patients (*M* = 4.43, *SD* = 0.12) than did public hospitals (*M* = 4.32, *SD* = 0.13). No significant differences (*p* = 0.629, Cohen’s *d* =  − 0.09) in patient experience were found between urban (*M* = 4.37, *SD* = 0.12) and rural (*M* = 4.37, *SD* = 0.14) hospitals.

### Proportion of future elective patients

Mixed model analysis using the yearly average patient experience as the independent variable, and the following year’s proportion of elective patients as the dependent variable, revealed a statistically significant effect of patient experience on the proportion of elective patients (β = 0.08; 95% confidence interval [CI] = 0.02–0.14; *t* [180.48] = 2.75; *p* = 0.007; AIC =  − 820.29; see Online Appendix, Table S3). Adjusting for covariates did not change this result, since the effect of yearly patient experience on the proportion of elective patients in the following year remained statistically significant (β = 0.09; 95% CI 0.03–0.14; *t* [174.91] = 2.93; *p* = 0.004; AIC =  − 829.95; see Table 1). When patient experience from the same year rather than the previous year was used, the effect size was smaller and no longer reached statistical significance (β = 0.01; *p* = 0.057; see Online Appendix, Table S4). A separate analysis of private hospitals revealed a significant, and even stronger, positive association between yearly patient experience and the proportion of elective patients in the following year (β = 0.17; 95% CI 0.06–0.27; *t* [67.70] = 3.04; *p* = 0.003; AIC =  − 282.50; see Online Appendix, Table S5). For public hospitals, the equivalent analysis showed a smaller, nonsignificant association (β = 0.04, *p* = 0.114; see Online Appendix, Table S6).

**Table 1:** Parameter estimates from mixed model regression, explaining the proportion of elective patients in all hospitals using the previous year’s patient experience

Parameter	Estimate	SE	*df*	*t*	*p*	95% CI	
						Lower bound	Upper bound
*Estimates of fixed effects*							
Intercept	0.04	0.14	209.02	0.27	0.787	− 0.23	0.31
PE_Lag	0.09	0.03	178.57	2.93	0.004	0.03	0.14
SI	0.24	0.08	215.84	2.87	0.004	0.078	0.41
Location	0.12	0.04	134.29	2.91	0.004	0.04	0.21
CMI	0.19	0.05	245.66	3.94	< 0.001	0.10	0.29
Discharges	− 8.21E−6	1.72E-6	158.90	− 4.77	< 0.001	− 1.16E−5	− 4.81E−6

PE_Lag = patient experience from the previous year (time-lagged), SI = supplemental health insurance (% of patients), Location = rural versus urban hospital location, CMI = case-mix index, and Discharges = number of acute-care discharges. Dependent variable: future proportion of elective patients. Sample: private and public hospitals

### Future revenue

When the full sample of Swiss hospitals was analyzed, no significant association was found between time-lagged patient experience and future revenue (β =  − 325.79; 95% CI  − 1456.11–804.53; *t* [301.23] =  − 0.57; *p* = 0.571; AIC = 5659.75; see Online Appendix, Table S7). However, a separate analysis of private hospitals identified a statistically significant, positive effect of the average yearly patient experience on the following year’s revenue (β =  − 257.86; 95% CI  − 1359.14–843.41; *t* [60.58] =  − 0.46; *p* = 0.030; AIC = 2256.51; see Online Appendix, Table S8). Once again, this result was not altered when covariates were added to the model (β = 1789.83; 95% CI 113.11–3466.55; *t* [61.13] = 2.13; *p* = 0.037; AIC = 2208.39; see Table 2), although the association disappeared if using patient experience from the same year rather than the previous year (β =  − 412.13, *p* = 0.617; see Online Appendix, Table S9). In contrast, the relationship between patient experience and future revenue was not significant if public hospitals were considered separately (β =  − 796.15, *p* = 0.068; see Online Appendix, Table S10).

**Table 2:** Parameter estimates from mixed model regression, explaining future revenue in private hospitals using the previous year’s patient experience

Parameter	Estimate	SE	*df*	*t*	*p*	95% CI	
						Lower bound	Upper bound
*Estimates of fixed effects*							
Intercept	1533.19	3870.37	64.08	0.40	0.693	− 6198.58	9264.96
PE_Lag	1789.83	838.55	61.13	2.13	0.037	113.11	3466.55
SI	2274.08	1177.93	51.16	1.93	0.059	− 90.53	4638.69
Location	309.99	686.91	36.43	0.45	0.654	− 1082.56	1702.54
CMI	− 789.43	1158.44	70.32	− 0.68	0.498	− 3099.69	1520.83
Discharges	− 0.05	0.06	40.63	− 0.78	0.439	− 0.16	0.07

PE_Lag = patient experience from the previous year (time-lagged), SI = supplemental health insurance (% of patients), Location = rural versus urban hospital location, CMI = case-mix index, and Discharges = number of acute-care discharges. Dependent variable: future revenue (standardized). Sample: private hospitals only

### Future costs

Yearly patient experience showed a significant negative effect on future costs (β =  − 1339.60; 95% CI  − 2273.71 to − 403.50; *t* [316.60] =  − 2.82; *p* = 0.005; AIC = 5587.85; see Online Appendix, Table S11). This finding was unchanged with inclusion of the covariates, in which case patient experience still displayed a significant negative relationship with future costs (β =  − 1191.13, 95% CI  − 2169.80 to − 212.46; *t* [321.58] =  − 2.39; *p* = 0.017; AIC = 5549.46; see Table 3). Similarly to previous analyses, the effect was smaller and nonsignificant if patient experience from the same year rather than the previous year was used (β =  − 615.17, *p* = 0.196; see Online Appendix, Table S12). Dividing the sample into private and public hospitals did not change the direction of the effect, although it no longer reached statistical significance for either private (β =  − 892.67, *p* = 0.357; see Online Appendix, Table S13) or public (β =  − 612.97, *p* = 0.268; see Online Appendix, Table S14) hospitals alone.

**Table 3:** Parameter estimates of mixed model regression, explaining future costs in all hospitals using the previous year’s patient experience

Parameter	Estimate	SE	*df*	*t*	*p*	95% CI	
						Lower bound	Upper bound
*Estimates of fixed effects*							
Intercept	14,157.86	2226.56	317.63	6.36	< 0.001	9777.18	18,538.54
PE_Lag	− 1191.13	497.45	321.58	− 2.39	0.017	− 2169.80	− 212.46
SI	269.24	562.60	117.68	0.48	0.633	− 844.89	1383.37
Location	235.94	243.57	97.37	0.97	0.335	− 247.46	719.34
CMI	− 273.49	455.50	116.74	− 0.60	0.549	− 1175.61	628.63
Discharges	0.01	0.01	104.17	1.37	0.173	− 0.01	0.04

PE_Lag = patient experience from the previous year (time-lagged), SI = supplemental health insurance (% of patients), Location = rural versus urban hospital location, CMI = case-mix index, and Discharges = number of acute-care discharges. Dependent variable: future costs (standardized). Sample: private and public hospitals

### Future profits

The yearly patient experience had a positive but nonsignificant effect on future profits (β = 870.88, 95% CI  − 122.34–1864.11; *t* [243.60] = 1.73; *p* = 0.085; AIC = 5655.98; see Online Appendix, Table S15). Inclusion of covariates did not affect this finding, with patient experience still showing a positive but nonsignificant association with future profits (β = 629.11, 95% CI  − 422.51–1680.73; *t* [292.33] = 1.18; *p* = 0.240; AIC = 5612.66; see Table 4). The effect was much smaller when using patient experience from the same year rather than the previous year (β = 109.77, *p* = 0.833; see Online Appendix, Table S16). Considering the time-lagged results for private and public hospitals separately, the association was positive for private hospitals (β = 1348.18, *p* = 0.216; see Online Appendix, Table S17) but negative for public hospitals (β =  − 111.85, *p* = 0.836; see Online Appendix, Table S18), although the effect did not reach statistical significance in either case.

**Table 4:** Parameter estimates of mixed model regression, explaining future profits in all hospitals using the previous year’s patient experience

Parameter	Estimate	SE	*df*	*t*	*p*	95% CI	
						Lower bound	Upper bound
*Estimates of fixed effects*							
Intercept	− 2485.87	2394.01	288.83	− 1.04	0.300	− 7197.78	2226.04
PE_Lag	629.11	534.33	292.33	1.18	0.240	− 422.51	1680.73
SI	1868.18	582.49	91.02	3.21	0.002	711.14	3025.21
Location	− 295.21	250.30	74.03	− 1.18	0.242	− 793.93	203.51
CMI	− 41.94	465.10	87.94	− 0.09	0.928	− 966.23	882.36
Discharges	0.01	0.01	78.41	0.54	0.590	− 0.02	0.03

PE_Lag = patient experience from the previous year (time-lagged), SI = supplemental health insurance (% of patients), Location = rural versus urban hospital location, CMI = case-mix index, and Discharges = number of acute-care discharges. Dependent variable: future profits (standardized). Sample: private and public hospitals

## Discussion

Better patient experience has previously been shown to be associated with higher patient loyalty. However, whether this translates into better financial performance of hospitals remains largely unknown to date. The goal of this study was therefore to comprehensively examine the relationship between patient experience and hospital financial performance, with regard to the proportion of elective patients, revenue, costs, and profits. We found that the average yearly patient experience was positively associated with the following year’s proportion of elective patients (in all hospitals) and revenue (in private hospitals), and negatively associated with the following year’s costs (in all hospitals), but not significantly associated with the next year’s profits (in all hospitals).

### Elective patients and future revenue

To the best of our knowledge, this study is the first to show that better patient experience is associated with a greater proportion of elective patients in the following (but not the current) year. These results have indirectly confirmed existing research regarding the association between patient experience and loyalty [7–9]; although in contrast to most previous studies, which only measured hypothetical constructs such as “intention to return” or “willingness to recommend,” we have supported the relationship using empirical evidence. Our results are also consistent with the findings of Fenton et al. [32], who showed that better patient experience was associated with enhanced utilization of hospital services but lower emergency use. One potential explanation is that patients with positive experiences tend to be more loyal and to recommend hospitals to others [10]. This may lead to a higher proportion of elective patients in the future, assuming that the number of emergency patients does not change because the acute nature of such admissions precludes them from freely choosing their hospital.

At any rate, elective patients are often more lucrative for hospitals than emergency patients, since they are more predictable and require less upfront service capacity [33]. For example, according to a study that analyzed the economic impact of elective versus non-elective surgeries during the COVID-19 pandemic, elective surgeries showed a five times higher net income compared to non-elective procedures in US hospitals [34]. Thus, a higher proportion of elective patients may be one reason for our finding that average yearly patient experience was positively associated with the following year’s revenue in private hospitals, as these hospitals focus particularly on elective patients. In Switzerland, a hospital’s revenue also depends on how many patients with supplemental health insurance it can treat, as these patients generate more revenue than those possessing only basic health insurance [3]. This effect was apparent in our analyses when focusing separately on public hospitals and private hospitals (where the proportion of patients with supplemental health insurance is generally higher), with a significant effect of patient experience on future revenue observed only for the latter. In pay-for-performance reimbursement systems, an improvement in patient experience is often rewarded with additional financial compensation [16]. However, since Switzerland does not provide such incentives, we were able to show an independent financial advantage of positive patient experience in terms of increased revenue.

### Future costs and profits

Our findings have also demonstrated an association between patient experience and hospital costs, with lower reported costs following higher previous year’s patient experience. This observation represents a novel contribution to the current research literature. It can be hypothesized that both direct and indirect effects are responsible for this negative association. On the one hand, our results agree with the assumption of Stelfox et al. [11] that better patient experience leads to fewer complaints and liability cases, which directly results in reduced costs. On the other hand, better patient experience is positively associated with clinical effectiveness and patient safety [35], which also reduces costs [36–38]. Another explanation may be found in the relationship between patient experience and employee satisfaction. Hospitals with satisfied employees have shown fewer medical errors and higher quality of care, as well as lower employee turnover rates [39, 40], both of which are associated with lower costs [36, 37, 41]. An association between employee satisfaction and patient experience has also been identified [42, 43]. Given the aforementioned findings, it seems reasonable to speculate that employee satisfaction and patient experience show a bidirectional relationship; or that employee satisfaction could even be the underlying cause of both better patient experience and reduced costs.

Regarding the influence of patient experience on profits, our results differ from those of Richter and Muhlestein [13], who demonstrated a positive effect of patient experience on profits and interpreted their findings in terms of patient loyalty and word-of-mouth recommendation. Most likely our conflicting findings just stem from an insufficient statistical power, as the point estimate of patient experience on future profits actually showed a large value (particularly for private hospitals) but did not reach the threshold of statistical significance due to an equally large standard error. A larger sample size of more than just 132 hospitals would presumably have confirmed the significant effects of patient experience on profits that were found in previous studies. Furthermore, the large standard errors presumably result from the great heterogeneity in Swiss hospitals. For example, the profits of Swiss hospitals are highly influence by the proportion of supplementally insured patients that the hospitals treat, as can be seen by the respective covariate in our model having a large effect on profits.

### Practical relevance, limitations, and future research

Our findings have demonstrated the importance of patient experience as both a management metric and a factor with potential financial consequences for hospitals. Specifically in private hospitals, patient experience may be an important indicator of patient loyalty, and hence, of future revenue and costs. However, this study also has several limitations, including our use of the case-mix indices (i.e., the sum of DRG cost weights) of hospitals to standardize their revenue, costs, and profits. Although this is a standard procedure in many instances, it must be noted that the DRG cost weights can be influenced by coding differences among hospitals. Assuming that private hospitals have higher incentives for upcoding would imply that a bias could have been introduced into our analyses by standardizing the financial measures. Another limitation may be the specific national setting in Switzerland, for two reasons. First, the number of hospitals is limited, which reduces statistical power. Second, the considerable heterogeneity of Swiss hospitals (e.g., with many special clinics providing specialized services) potentially restricts the generalizability of our findings. Future research should aim to replicate our results in different national settings with a larger sample of hospitals to investigate the complex relationship between patient experience and profits more closely. Particular attention could be given to the relationship between patient experience and elective patients, or that between patient experience and employee satisfaction; or to investigation of causality with regard to patient experience and financial performance.

## Conclusion

In this study, we examined the associations between patient experience and financial performance of hospitals. We found that better patient experience in a given year was linked to a higher proportion of elective patients and greater revenue in the following year (with the latter particularly applying to private hospitals). In addition, better patient experience was associated with lower costs in the following year. Hospital managers and regulators may leverage these findings to identify strategies to increase revenue and lower costs, or to gain new insight into the underlying mechanisms linking patient experience and financial performance.

### Supplementary Information

Below is the link to the electronic supplementary material.

Supplementary file 1 (DOCX 137 KB)

## Data Availability

The data that support the findings of this study are openly available: Federal Office of Public Health (FOPH) at https://www.bag.admin.ch/bag/de/home/zahlen-und-statistiken/zahlen-fakten-zu-spitaelern/kennzahlen-der-schweizer-spitaeler.html; Swiss National Association for Quality Development in Hospitals and Clinics (ANQ) at https://www.anq.ch/de/fachbereiche/akutsomatik/messergebnisse-akutsomatik/step2/measure/1. As the process of data extraction and matching from two different sources is time consuming, a dataset containing the combined raw data can be obtained directly from the corresponding author (AG) upon request.
