# Supplementary material for: Patient-reported experience is associated with higher future revenue and lower costs of hospitals
Source: Eur J Health Econ. 2023 Dec 9;25(6):1031–9. doi: 10.1007/s10198-023-01646-y (PMC11283410; doi:10.1007/s10198-023-01646-y)
Supplement: Supplementary file 1 — (DOCX 137 KB) [file 10198_2023_1646_MOESM1_ESM.docx]

## Appendix

**Table S1**. ^a^ Questions included in the ANQ national patient experience survey.

| **ANQ question** | **Type of question**^a^ |
| --- | --- |
| 1. How would you rate the quality of your treatment (provided by the doctors and nursing staff)? | Patient satisfaction |
| 1. Did you have the opportunity to ask questions? | Patient experience |
| 1. Did you receive clear answers to your questions? | Patient experience |
| 1. Did you receive a clear explanation regarding the purpose of the medication that you were required to take after you returned home? | Patient experience |
| 1. How would you rate the organization of your discharge? | Patient experience |

^a^Note: The assessment of the type of question was made by the authors.

**Table S2**.^a^ Descriptive analysis of the questions from the ANQ national patient experience survey.

|  | Year | | | | | | | |
| --- | --- | --- | --- | --- | --- | --- | --- | --- |
|  | 2016  *n* = 111 | | 2017  *n* = 113 | | 2018  *n* = 128 | | 2019  *n* = 128 | |
|  | *M* | *SD* | *M* | *SD* | *M* | *SD* | *M* | *SD* |
| Q1: Quality of care | 4.21 | 0.16 | 4.23 | 0.14 | 4.23 | 0.17 | 4.20 | 0.16 |
| Q2: Opportunity to ask questions | 4.52 | 0.20 | 4.53 | 0.18 | 4.52 | 0.22 | 4.52 | 0.19 |
| Q3: Clear answers | 4.57 | 0.14 | 4.56 | 0.14 | 4.57 | 0.15 | 4.56 | 0.14 |
| Q4: Purpose of medication | 4.51 | 0.15 | 4.50 | 0.13 | 4.51 | 0.13 | 4.48 | 0.16 |
| Q5: Discharge organization | 4.01 | 0.18 | 4.03 | 0.16 | 4.04 | 0.19 | 4.00 | 0.21 |
| Patient experience (total) | 4.36 | 0.15 | 4.37 | 0.13 | 4.37 | 0.14 | 4.35 | 0.13 |

^a^Note: Q = question

**Figure S1**. (a) Histogram showing the distribution of elective patients across the whole sample. (b) Population pyramid comparing the distribution of elective patient proportion (%) between private and public hospitals (indicated by red and blue bars, respectively).

| (a) | (b) |
| --- | --- |
| 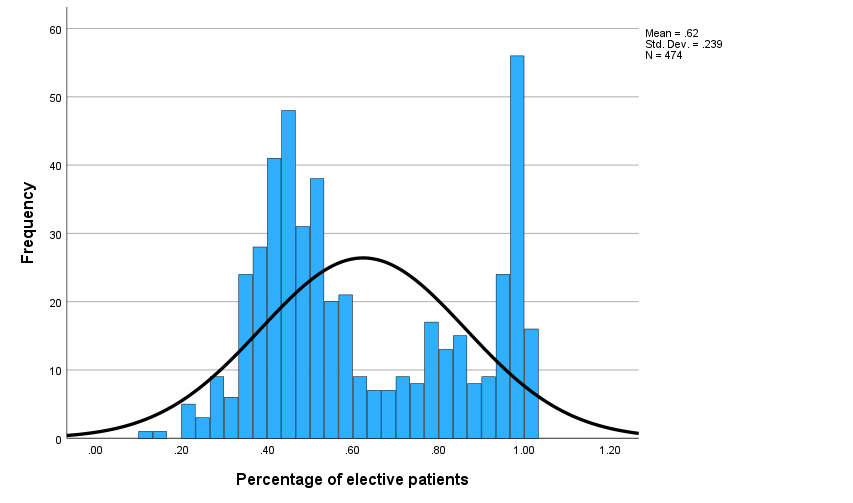 | 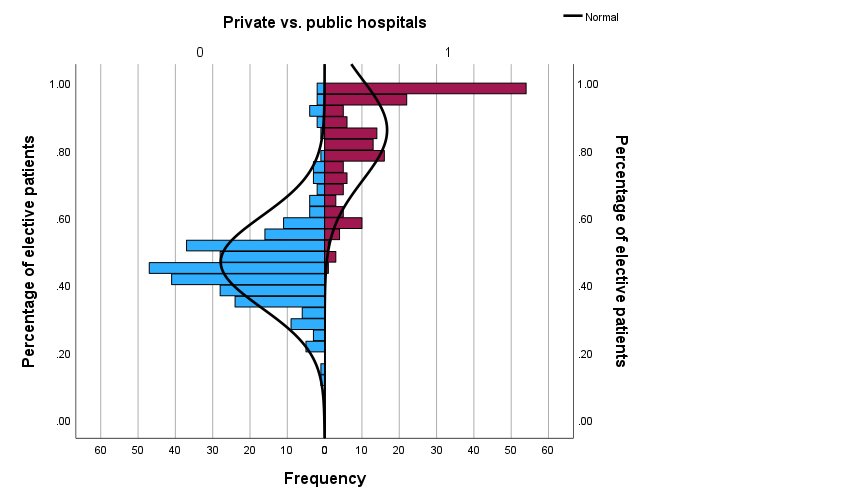 |

**Proportion of future elective patients**

**Table S3**.^a^ Parameter estimates from mixed model regression, explaining the proportion of future elective patients in all hospitals using the previous year’s patient experience (without covariates).

| *Estimates of fixed effects* | | | | | | | |
| --- | --- | --- | --- | --- | --- | --- | --- |
| Parameter | Estimate | Std. Error | df | t | *p* | 95% CI | |
|  |  |  |  |  |  | Lower Bound | Upper Bound |
| Intercept | 0.28 | 0.13 | 190.65 | 2.21 | .028 | 0.03 | 0.52 |
| PE_Lag | 0.08 | 0.03 | 180.49 | 2.75 | .007 | 0.02 | 0.14 |
| ^a^Note: PE_Lag = patient experience from the previous year (time-lagged). Dependent variable: future proportion of elective patients. Sample: private and public hospitals. | | | | | | | |

**Table S4**.^a^ Parameter estimates from mixed model regression, explaining the proportion of future elective patients in all hospitals using the same year’s patient experience.

| *Estimates of fixed effects* | | | | | | | |
| --- | --- | --- | --- | --- | --- | --- | --- |
| Parameter | Estimate | Std. Error | df | t | *p* | 95% CI | |
|  |  |  |  |  |  | Lower Bound | Upper Bound |
| Intercept | 0.46 | 0.11 | 263.19 | 4.05 | < .001 | 0.23 | 0.68 |
| PE | 0.01 | 0.02 | 194.53 | 0.62 | .534 | −0.03 | 0.06 |
| SI | 0.20 | 0.08 | 301.23 | 2.60 | .010 | 0.05 | 0.35 |
| Location | 0.13 | 0.04 | 134.79 | 2.95 | .004 | 0.04 | 0.21 |
| CMI | 0.09 | 0.05 | 353.59 | 1.82 | .069 | −0.01 | 0.18 |
| Discharges | −6.87E−6 | 1.73E−6 | 179.33 | −3.98 | < .001 | −1.027E−5 | −3.4E−6 |
| ^a^Note: PE = patient experience from the same year (not time-lagged), SI = supplemental health insurance (% of patients), Location = rural vs. urban hospital location, CMI = case-mix index, and Discharges = number of acute-care discharges. Dependent variable: future proportion of elective patients. Sample: private and public hospitals. | | | | | | | |

**Table S5**.^a^ Parameter estimates from mixed model regression, explaining the proportion of future elective patients in private hospitals using the previous year’s patient experience.

| *Estimates of fixed effects* | | | | | | | |
| --- | --- | --- | --- | --- | --- | --- | --- |
| Parameter | Estimate | Std. Error | df | t | *p* | 95% CI | |
|  |  |  |  |  |  | Lower Bound | Upper Bound |
| Intercept | 0.01 | 0.25 | 72.75 | 0.04 | .965 | −0.49 | 0.51 |
| PE_Lag | 0.17 | 0.05 | 67.70 | 3.04 | .003 | 0.06 | 0.27 |
| SI | 0.06 | 0.08 | 59.13 | 0.75 | .455 | −0.10 | 0.21 |
| Location | 0.01 | 0.04 | 49.82 | 0.15 | .882 | −0.08 | 0.10 |
| CMI | 0.14 | 0.06 | 86.22 | 2.31 | .024 | 0.02 | 0.26 |
| Discharges | −9.52E−6 | 3.68E−6 | 60.59 | −2.59 | .012 | −1.69E−5 | −2.17E−6 |
| ^a^Note: PE_Lag = patient experience from the previous year (time-lagged), SI = supplemental health insurance (% of patients), Location = rural vs. urban hospital location, CMI = case-mix index, and Discharges = number of acute-care discharges. Dependent variable: future proportion of elective patients. Sample: private hospitals only. | | | | | | | |
|  | | | | | | | |

**Table S6**.^a^ Parameter estimates from mixed model regression, explaining the proportion of future elective patients in public hospitals using the previous year’s patient experience.

| *Estimates of fixed effects* | | | | | | | |
| --- | --- | --- | --- | --- | --- | --- | --- |
| Parameter | Estimate | Std. Error | df | t | *p* | 95% CI | |
|  |  |  |  |  |  | Lower Bound | Upper Bound |
| Intercept | 0.14 | 0.13 | 173.08 | 1.06 | .290 | −0.12 | 0.40 |
| PE_Lag | 0.04 | 0.03 | 142.55 | 1.59 | .114 | −0.01 | 0.10 |
| SI | 0.00 | 0.16 | 167.60 | .01 | .994 | −0.32 | 0.32 |
| Location | 0.12 | 0.04 | 79.78 | 2.84 | .006 | 0.03 | 0.20 |
| CMI | 0.13 | 0.06 | 170.61 | 2.07 | .040 | 0.01 | 0.25 |
| Discharges | −2.08E−6 | 1.39E−6 | 89.78 | −1.50 | .137 | −4.83E−6 | 6.72E−7 |
| ^a^Note: PE_Lag = patient experience from the previous year (time-lagged), SI = supplemental health insurance (% of patients), Location = rural vs. urban hospital location, CMI = case-mix index, and Discharges = number of acute-care discharges. Dependent variable: future proportion of elective patients. Sample: public hospitals only. | | | | | | | |

**Future revenue**

**Table S7**.^a^ Parameter estimates from mixed model regression, explaining the future revenue in all hospitals using the previous year’s patient experience.

| *Estimates of fixed effects* | | | | | | | |
| --- | --- | --- | --- | --- | --- | --- | --- |
| Parameter | Estimate | Std. Error | df | t | *p* | 95% CI | |
|  |  |  |  |  |  | Lower Bound | Upper Bound |
| Intercept | 10,810.28 | 2,585.78 | 305.35 | 4.18 | < .001 | 5,722.08 | 15,898.48 |
| PE_Lag | −325.79 | 574.39 | 301.23 | −0.57 | .571 | −1,456.11 | 804.53 |
| SI | 2,360.60 | 753.49 | 117.34 | 3.13 | .002 | 868.39 | 3,852.81 |
| Location | −76.80 | 331.60 | 96.01 | −0.23 | .817 | −735.01 | 581.42 |
| CMI | −585.57 | 602.37 | 129.24 | −0.97 | .333 | −1,777.35 | 606.20 |
| Discharges | 0.02 | 0.01 | 102.45 | 1.70 | .092 | −0.00 | 0.05 |
| ^a^Note: PE_Lag = patient experience from the previous year (time-lagged), SI = supplemental health insurance (% of patients), Location = rural vs. urban hospital location, CMI = case-mix index, and Discharges = number of acute-care discharges. Dependent variable: future revenue (standardized). Sample: private and public hospitals. | | | | | | | |

**Table S8**.^a^ Parameter estimates from mixed model regression, explaining the future revenue in private hospitals using the previous year’s patient experience (without covariates).

| *Estimates of fixed effects* | | | | | | | |
| --- | --- | --- | --- | --- | --- | --- | --- |
| Parameter | Estimate | Std. Error | df | t | *p*-value | 95% CI | |
|  |  |  |  |  |  | Lower Bound | Upper Bound |
| Intercept | 10,712.18 | 2,454.32 | 310.18 | 4.37 | < .001 | 5,882.97 | 15,541.40 |
| PE_Lag | −257.86 | 559.69 | 309.50 | −0.46 | .645 | −1,359.14 | 843.41 |
| ^a^Note: PE_Lag = patient experience from the previous year (time-lagged). Dependent variable: future revenue (standardized). Sample: private hospitals only. | | | | | | | |

**Table S9.** ^a^ Parameter estimates from mixed model regression, explaining the future revenue in private hospitals using the same year’s patient experience.

| *Estimates of fixed effects* | | | | | | | |
| --- | --- | --- | --- | --- | --- | --- | --- |
| Parameter | Estimate | Std. Error | df | t | *p* | 95% CI | |
|  |  |  |  |  |  | Lower Bound | Upper Bound |
| Intercept | 11,729.56 | 3,891.06 | 97.59 | 3.01 | .003 | 4,007.47 | 19,451.65 |
| PE | -412.13 | 821.60 | 85.33 | -0.50 | .617 | -2,045.59 | 1,221.33 |
| SI | 2,879.75 | 1,203.47 | 76.44 | 2.39 | .019 | 483.04 | 5,276.45 |
| Location | 416.05 | 700.38 | 54.03 | 0.59 | .555 | -988.12 | 1,820.21 |
| CMI | -1,114.21 | 1,113.04 | 107.38 | -1.00 | .319 | -3,320.60 | 1,092.17 |
| Discharges | -0.10 | 0.06 | 63.12 | -1.63 | .109 | -0.21 | 0.02 |
| Note. PE = patient experience from the same year (not time-lagged), SI = supplemental health insurance (% of patients), Location = rural vs. urban hospital location, CMI = case-mix index, and Discharges = number of acute-care discharges. Dependent variable: future revenue (standardized). Sample: private hospitals only. | | | | | | | |

**Table S10**.^a^ Parameter estimates from mixed model regression, explaining the future revenue in public hospitals using the previous year’s patient experience.

| *Estimates of fixed effects* | | | | | | | |
| --- | --- | --- | --- | --- | --- | --- | --- |
| Parameter | Estimate | Std. Error | df | T | *p* | 95% CI | |
|  |  |  |  |  |  | Lower Bound | Upper Bound |
| Intercept | 12,108.94 | 1,966.79 | 155.09 | 6.16 | < .001 | 8,223.79 | 15,994.08 |
| PE_Lag | −796.15 | 433.76 | 148.35 | −1.84 | .068 | −1,653.29 | 60.98 |
| SI | 2,407.44 | 1,421.76 | 97.49 | 1.69 | .094 | −414.19 | 5,229.06 |
| Location | −195.85 | 286.63 | 72.66 | −0.68 | .497 | −767.15 | 375.44 |
| CMI | 625.83 | 530.83 | 87.42 | 1.18 | .242 | −429.17 | 1,680.84 |
| Discharges | 0.01 | 0.01 | 75.56 | 1.07 | .290 | −0.01 | 0.03 |
| ^a^Note: PE_Lag = patient experience from the previous year (time-lagged), SI = supplemental health insurance (% of patients), Location = rural vs. urban hospital location, CMI = case-mix index, and Discharges = number of acute-care discharges. Dependent variable: future revenues (standardized). Sample: public hospitals only. | | | | | | | |

**Future costs**

**Table S11**.^a^ Parameter estimates from mixed model regression, explaining the future costs in all hospitals using the previous year’s patient experience (without covariates).

| *Estimates of fixed effects* | | | | | | | |
| --- | --- | --- | --- | --- | --- | --- | --- |
| Parameter | Estimate | Std. Error | df | t | *p* | 95% CI | |
|  |  |  |  |  |  | Lower Bound | Upper Bound |
| Intercept | 14,830.99 | 2,081.94 | 315.88 | 7.12 | < .001 | 10,734.77 | 18,927.20 |
| PE_Lag | −1,338.60 | 475.28 | 316.60 | −2.82 | .005 | −2,273.71 | −403.50 |
| ^a^Note: PE_Lag = patient experience from the previous year (time-lagged). Dependent variable: future costs (standardized). Sample: private and public hospitals. | | | | | | | |

**Table S12**.^a^ Parameter estimates from mixed model regression, explaining the future costs in all hospitals using the same year’s patient experience.

| *Estimates of fixed effects* | | | | | | | |
| --- | --- | --- | --- | --- | --- | --- | --- |
| Parameter | Estimate | Std. Error | df | t | *p* | 95% CI | |
|  |  |  |  |  |  | Lower Bound | Upper Bound |
| Intercept | 11,935.36 | 2,167.92 | 362.37 | 5.51 | < .001 | 7,672.07 | 16,198.64 |
| PE | −615.17 | 475.05 | 340.21 | −1.30 | .196 | −1,549.58 | 319.25 |
| SI | 144.08 | 687.37 | 121.61 | 0.21 | .834 | −1,216.69 | 1,504.84 |
| Location | 269.33 | 319.02 | 104.32 | 0.84 | .400 | −363.27 | 901.94 |
| CMI | −372.35 | 578.31 | 148.89 | −0.64 | .521 | −1,515.10 | 770.39 |
| Discharges | 0.01 | 0.01 | 110.40 | 0.68 | .496 | −0.02 | 0.04 |
| ^a^Note: PE = patient experience from the same year (not time-lagged), SI = supplemental health insurance (% of patients), Location = rural vs. urban hospital location, CMI = case-mix index, and Discharges = number of acute-care discharges. Dependent variable: future costs (standardized). Sample: private and public hospitals. | | | | | | | |

**Table S13**.^a^ Parameter estimates from mixed model regression, explaining the future costs in private hospitals using the previous year’s patient experience.

| *Estimates of fixed effects* | | | | | | | |
| --- | --- | --- | --- | --- | --- | --- | --- |
| Parameter | Estimate | Std. Error | df | t | *p* | 95% CI | |
|  |  |  |  |  |  | Lower Bound | Upper Bound |
| Intercept | 11,968.07 | 4,374.73 | 120.50 | 2.74 | .007 | 3,306.76 | 20,629.38 |
| PE_Lag | −892.66 | 964.82 | 120.10 | −0.93 | .357 | −2,802.92 | 1,017.60 |
| SI | 1,167.60 | 741.16 | 52.37 | 1.58 | .121 | −319.40 | 2,654.59 |
| Location | 524.68 | 406.83 | 48.05 | 1.29 | .203 | −293.29 | 1,342.65 |
| CMI | −155.87 | 782.25 | 59.39 | −0.20 | .843 | −1,720.94 | 1,409.20 |
| Discharges | 0.01 | 0.04 | 47.67 | 0.31 | .758 | −0.06 | 0.08 |
| ^a^Note: PE_Lag = patient experience from the previous year (time-lagged), SI = supplemental health insurance (% of patients), Location = rural vs. urban hospital location, CMI = case-mix index, and Discharges = number of acute-care discharges. Dependent variable: future costs (standardized). Sample: private hospitals only. | | | | | | | |

**Table S14**.^a^ Parameter estimates from mixed model regression, explaining the future costs in public hospitals using the previous year’s patient experience.

| *Estimates of fixed effects* | | | | | | | |
| --- | --- | --- | --- | --- | --- | --- | --- |
| Parameter | Estimate | Std. Error | df | t | *p* | 95% CI | |
|  |  |  |  |  |  | Lower Bound | Upper Bound |
| Intercept | 11,800.05 | 2,478.45 | 198.09 | 4.76 | < .001 | 6,912.51 | 16,687.59 |
| PE_Lag | −612.97 | 551.48 | 200.62 | −1.11 | .268 | −1,700.42 | 474.48 |
| SI | −2,069.19 | 1,695.73 | 73.55 | −1.22 | .226 | −5,448.35 | 1,309.96 |
| Location | 117.00 | 329.66 | 50.45 | 0.36 | .724 | −544.99 | 778.98 |
| CMI | 520.90 | 620.37 | 61.57 | 0.84 | .404 | −719.38 | 1,761.17 |
| Discharges | −0.01 | 0.01 | 53.92 | −0.40 | .691 | −0.03 | 0.02 |
| ^a^Note: PE_Lag = patient experience from the previous year (time-lagged), SI = supplemental health insurance (% of patients), Location = rural vs. urban hospital location, CMI = case-mix index, and Discharges = number of acute-care discharges. Dependent variable: future costs (standardized). Sample: public hospitals only. | | | | | | | |

**Future profits**

**Table S15**.^a^ Parameter estimates from mixed model regression, explaining the future profits in all hospitals using the previous year’s patient experience (without covariates).

| *Estimates of fixed effects* | | | | | | | |
| --- | --- | --- | --- | --- | --- | --- | --- |
| Parameter | Estimate | Std. Error | df | t | *p* | 95% CI | |
|  |  |  |  |  |  | Lower Bound | Upper Bound |
| Intercept | −3,196.96 | 2,209.16 | 241.95 | −1.45 | .149 | −7,548.60 | 1,154.67 |
| PE_Lag | 870.88 | 504.24 | 243.60 | 1.73 | .085 | −122.34 | 1,864.11 |
| ^a^Note: PE_Lag = patient experience from the previous year (time-lagged). Dependent variable: future profits (standardized). Sample: private and public hospitals. | | | | | | | |

**Table S16**.^a^ Parameter estimates from mixed model regression, explaining the future profits in all hospitals using the same year’s patient experience.

| *Estimates of fixed effects* | | | | | | | |
| --- | --- | --- | --- | --- | --- | --- | --- |
| Parameter | Estimate | Std. Error | df | t | *p* | 95% CI | |
|  |  |  |  |  |  | Lower Bound | Upper Bound |
| Intercept | −257.47 | 2,359.82 | 337.37 | −0.11 | .913 | −4,899.28 | 4,384.35 |
| PE | 109.77 | 520.40 | 327.58 | 0.21 | .833 | −913.98 | 1,133.53 |
| SI | 2,319.41 | 712.22 | 135.18 | 3.26 | .001 | 910.88 | 3,727.93 |
| Location | −172.12 | 316.48 | 118.04 | −0.54 | .588 | −798.83 | 454.59 |
| CMI | −298.90 | 578.73 | 151.44 | −0.52 | .606 | −1,442.31 | 844.52 |
| Discharges | 0.01 | 0.01 | 124.49 | 0.78 | .438 | −0.02 | 0.04 |
| ^a^Note: PE = patient experience from the same year (not time-lagged), SI = supplemental health insurance (% of patients), Location = rural vs. urban hospital location, CMI = case-mix index, and Discharges = number of acute-care discharges. Dependent variable: future profits (standardized). Sample: private and public hospitals. | | | | | | | |

**Table S17**.^a^ Parameter estimates from mixed model regression, explaining the future profits in private hospitals using the previous year’s patient experience.

| *Estimates of fixed effects* | | | | | | | |
| --- | --- | --- | --- | --- | --- | --- | --- |
| Parameter | Estimate | Std. Error | df | t | *p* | 95% CI | |
|  |  |  |  |  |  | Lower Bound | Upper Bound |
| Intercept | −4,480.15 | 4,929.34 | 107.95 | −0.91 | .365 | −14,251.02 | 5,290.71 |
| PE_Lag | 1,348.18 | 1,082.55 | 109.63 | 1.25 | .216 | −797.27 | 3,493.63 |
| SI | 1,557.58 | 860.49 | 26.46 | 1.81 | .082 | −209.68 | 3,324.84 |
| Location | −301.75 | 474.31 | 23.48 | −0.64 | .531 | −1,281.82 | 678.32 |
| CMI | −842.53 | 888.27 | 29.96 | −0.95 | .350 | −2,656.74 | 971.68 |
| Discharges | −0.04 | 0.04 | 22.17 | −0.91 | .371 | −0.12 | 0.05 |
| ^a^Note: PE_Lag = patient experience from the previous year (time-lagged), SI = supplemental health insurance (% of patients), Location = rural vs. urban hospital location, CMI = case-mix index, and Discharges = number of acute-care discharges. Dependent variable: future profits (standardized). Sample: private hospitals only. | | | | | | | |

**Table S18**.^a^ Parameter estimates from mixed model regression, explaining the future profits in public hospitals using the previous year’s patient experience.

| *Estimates of fixed effects* | | | | | | | |
| --- | --- | --- | --- | --- | --- | --- | --- |
| Parameter | Estimate | Std. Error | df | t | *p* | 95% CI | |
|  |  |  |  |  |  | Lower Bound | Upper Bound |
| Intercept | 12.47 | 2,411.35 | 184.77 | 0.01 | .996 | −4,744.85 | 4,769.80 |
| PE_Lag | −111.85 | 540.78 | 192.36 | −0.21 | .836 | −1,178.48 | 954.78 |
| SI | 4,277.23 | 1,437.06 | 74.65 | 2.98 | .004 | 1,414.24 | 7,140.21 |
| Location | −202.61 | 269.21 | 51.86 | −0.75 | .455 | −742.86 | 337.64 |
| CMI | 105.48 | 517.41 | 60.71 | 0.20 | .839 | −929.25 | 1,140.21 |
| Discharges | 0.01 | 0.01 | 56.48 | 1.35 | .181 | −0.01 | 0.03 |
| ^a^Note: PE_Lag = patient experience from the previous year (time-lagged), SI = supplemental health insurance (% of patients), Location = rural vs. urban hospital location, CMI = case-mix index, and Discharges = number of acute-care discharges. Dependent variable: future profits (standardized). Sample: public hospitals only. | | | | | | | |
